# Arab community perceptions and awareness of family medicine: a systematic review

**DOI:** 10.3399/BJGPO.2024.0104

**Published:** 2025-03-10

**Authors:** Beesan Maraqa, Zaher Nazzal, Therese Zink

**Affiliations:** 1 College of Medicine, Hebron University, Hebron, Palestine; 2 Department of Medicine, Faculty of Medicine, An-Najah National University, Nablus, Palestine; 3 Brown University, Rhode Island, United States

**Keywords:** family medicine, attitude, community perception, general practice, systematic review

## Abstract

**Background:**

Family medicine (FM), often known as general practice, is the foundation of sustainable and universal healthcare worldwide. As a new specialty in the Eastern Mediterranean Region (EMR), it must recruit doctors and gain public acceptability, which has traditionally favoured specialists.

**Aim:**

This research examined studies on Arab populations' attitudes towards FM to discover the barriers to creating and embracing this vital specialty.

**Design & setting:**

This review was based on the Preferred Reporting Items for Systematic Reviews and Meta-Analyses (PRISMA) guidelines and encompassed peer-reviewed articles from reputable sources such as PsycNet, Web of Science, PubMed, Embase, Scopus, and grey literature.

**Method:**

A comprehensive search was conducted across databases for peer-reviewed studies that explored Arabs' awareness, perceptions, and attitudes towards FM and physicians.

**Results:**

After a rigorous selection process, 19 studies were deemed suitable for analysis. These studies encompassed diverse participants, including medical students, physicians, patients, and the general public. The overall perception of FM was positive, but it was noted that few had direct exposure to family physicians during their medical education or in the clinical setting.

**Conclusion:**

Our review findings suggest the following five recommendations: (1) an education campaign for the general public about the role of FM; (2) increasing training capacity for family physicians; (3) early exposure to family physicians during medical school; (4) developing a process for continually improving the education and quality of family physicians; and (5) further research on the challenges to FM practice in Arab countries to understand the situation better and work toward its improvement.

## How this fits in

Historically, the public and medical students in the Eastern Mediterranean Region (EMR) have favoured care from specialists. As a new specialty in EMR, family medicine (FM) must recruit medical students and residents to choose the specialty. The public must understand the value FM adds to health care. To expand the acceptability of FM by these populations, this review suggests the following: (1) an education campaign for the general public; (2) increasing training capacity for family physicians; (3) early exposure to family physicians during medical school; (4) developing a process for continually improving the education and quality of family physician practice so that they can incorporate the rapid changes in science into the care they provide; (5) further research.

## Introduction

Family medicine (FM), often known as general practice in some countries, is the basis of healthcare systems worldwide. It is regarded as the first point of contact for comprehensive, continuous, and coordinated community-based practice that ensures healthcare quality and equity.^1^ FM is a specialty in primary health care (PHC) and is considered a cornerstone of a sustainable health system. FM cares for all ages, addressing preventive care and managing chronic illnesses. PHC is the most inclusive, effective, and efficient way to improve people’s physical and mental health and social wellbeing.^2^ Most countries incorporate the FM specialty into their PHC as populations age and face considerable ongoing lifestyle, equity, and chronic illness concerns.^3^

FM is a relatively new discipline in low-income countries. A period of expansion followed, which included the addition of community practices, medical school courses, residency programmes, and organisational adaptations to empower PHC as early as the 1970s.^4^ In the Eastern Mediterranean Region (EMR), FM started in the early 1980s and has advanced slowly in most Arab countries compared with other clinical medical specialties.^5^ A review of FM in 22 countries of the EMR between 2014 and 2016, which was conducted by the World Health Organization (WHO), found that family practice is included in the national health policies of 16 countries (72%), with 13 countries planning to expand it. The review revealed a severe lack of family physicians in the EMR, requiring immediate action. The population density of family physicians ranges from 1.84 per 10 000 to 0.31 per 100 000, significantly below WHO standards, and the production of family doctors fluctuates despite relatively solid political support for family practice programmes.^6^ The scarcity of doctors to fill open positions has shifted the focus of political and media attention to FM.^7^ It’s worth noting that most of the doctors providing PHC in the EMR are GPs, representing medical doctors (MDs) who have graduated from medical school and completed a rotating hospital-based internship without any further residency programme in FM.

Ignoring the value of FM and its importance to the rest of the health system and society impedes FM development. FM is gaining recognition in some Arab countries but also confronting specific challenges, such as the dominance of specialist care, varying levels of awareness about the role of family physicians, and differing perceptions of primary healthcare quality.^5^ Other issues include low professional prestige for family physicians, detrimental policies, and apparent signs of discrimination.^8^ Population-based initiatives effectively bring about social change against FM discrimination and support the importance of such medical practice to the community.^9^

Numerous studies have focused on primary care and family physicians, highlighting their benefits and impact.^10^ Patients tend to bypass PHC centres in favour of specialised medical centres and tertiary institutions, even when it may not be necessary.^11^ The Arab population has preferred specialised care over FM, possibly owing to a perceived lack of trust in FM physicians' knowledge and competence.^12,13^

The accessibility of reliable information regarding the Arab community’s awareness and perception of FM as an integral component in PHC may be limited owing to many factors. Generally, funding and research priorities dictate the extent of an investigation. In addition, government regulations and privacy concerns may limit data accessibility, particularly at the community level. Access to comprehensive data on community attitudes may consequently be impeded. Moreover, Arab communities exhibit remarkable diversity, which is evident in the variations observed in socioeconomic status, healthcare systems, and cultural practices. This diversity must be accounted for in research if a comprehensive understanding is to be attained; however, this can present logistical challenges. This research aimed to examine studies investigating community perceptions of FM in Arab countries to identify obstacles that hinder the development and adoption of this crucial practice in Arab countries.

## Method

This systematic review followed the Preferred Reporting Items for Systematic Reviews and Meta-Analyses (PRISMA) guidelines.^14^ A search of the PROSPERO database revealed no prior protocol for this review.

### Eligibility criteria

Inclusion criteria included all published, peer-reviewed, and any language research between database inception and 20 April 2024. The sample population was mandated to consist of individuals from the Arab community, irrespective of their occupation, which could include medical students and dental students, among others. The review included publications reporting the awareness of, perceptions of, and attitudes towards FM and family physicians in the following Arab countries: Egypt; Bahrain; Iraq; Jordan; Kuwait; Lebanon; Libya; Morocco; Oman; Saudi Arabia; Sudan; Syria; Tunisia; United Arab Emirates; Yemen; Algeria; occupied Palestinian territories; Sudan; Republic of the Sudan; Mauritania; and Comoros Islands. Observational studies (cross-sectional, cohort, and case-control), qualitative designs, and systematic reviews were all included in the search. The article was required to be a primary study or research report. Abstracts and posters were used, but editorials, commentaries, and books were excluded.

### Search strategy

An electronic search was undertaken of PubMed, Embase, Web of Science, Scopus, and PsycNet to identify all relevant studies published in peer-reviewed journals. No restrictions were imposed on the language search. Grey literature was examined by locating FM-related organisations in the region and checking their websites for answers to our study topic. The keywords 'Physicians”, “Family”, 'Family Practice', ”General Practice”, “Awareness”, “Attitude”, “Perception”, ”Public Opinion”, “Egypt”, “Bahrain”, “Qatar”, “Iraq”, “Jordan”, “Kuwait”, “Lebanon”, “Libya”, “Morocco”, “Oman”, ”Middle East“, “Saudi Arabia”, “Sudan”, “Syria”, “Syrian Arab Republic”, “Tunisia”, “United Arab Emirates”, “Yemen”, “Algeria”, “West Bank”, “Palestine”, “Gaza strip”, “Sudan”, “republic of the Sudan”, “Mauritania”, and “Comoros Island”, were combined using Boolean operators ('AND' and 'OR').

### Eligibility screening

Article details were imported into Mendeley, exported to Covidence (https://www.covidence.org), and duplicates removed. Two authors (BM, ZN) independently screened all titles, abstracts, and articles according to the eligibility criteria; disagreements were resolved by consensus between the two authors.

### Data collection process

A comprehensive codebook was developed as per Cooper guidelines.^15^ Two authors (BM, ZN) extracted data from eligible studies. Categories of coded variables included study identification, research design, funding, source of the article, population features (target population, location, sample size, response rate of surveys), outcomes (the perceptions of the Arab community regarding FM, family practice, and PHC practice in the Arab World, Arab community understanding of the role of the family physician, and the Arab community attitude towards family practice and the FM specialty.

### Risk of bias

Two investigators independently assessed the methodological quality of each included cross-sectional study using a quality assessment tool for cross-sectional studies.^16^ Any disagreement on the quality assessment checklist was resolved by consultation with a third reviewer. The evaluation instrument assessed the sample’s representativeness, sample size and technique, non-response bias, and the acceptability of the measurement instrument. The total score ranged between 0 and 9. Studies scoring 0–3 points were deemed to have a low risk of bias, whereas studies scoring 4–6 points were considered moderate, and studies scoring 7–9 points were deemed to have a high risk of bias. The overall quality of the study was classified as high, moderate, or low.

### Qualitative study assessment

The qualitative studies were assessed using a qualitative quality assessment instrument. The present tool was sourced from Hawker *et al*'s research^17^and consists of nine questions that can each be answered 'good', 'fair', 'poor', or 'very poor'. After using the tool on the studies, we converted the results into a numerical score by giving the answers a range of 1 (very poor) to 4 (good) points. Each study resulted in a minimum of nine points and a maximum of 36 points. The scores correspond to 30–36 points for high quality, 24–29 for medium quality, and 0–19 for low quality.

### Data extraction

A data extraction form was created to collect the information required for data synthesis. Pilot tests were carried out before the implementation of this form. Two reviewers extracted the data and analysed it to attain a deeper understanding of the participants' viewpoints toward family medicine, which varied depending on the study’s objectives, ranging from patient satisfaction in centres run by family doctors versus centres run by a GP; and a medical doctor with no formal training in FM to medical students' knowledge and awareness of FM as a specialty. The findings were presented in two separate tables, the first of which included the titles, country of study, goal and study design, sample size, and time of study execution, as well as the net quality. The second represented the study’s inclusion criteria and the study’s outcome. For this paper, FM refers to physicians specialising in FM, and GPs are medical school graduates who complete a year of rotating hospital clerkships without training in FM.

## Results

**Figure 1.**
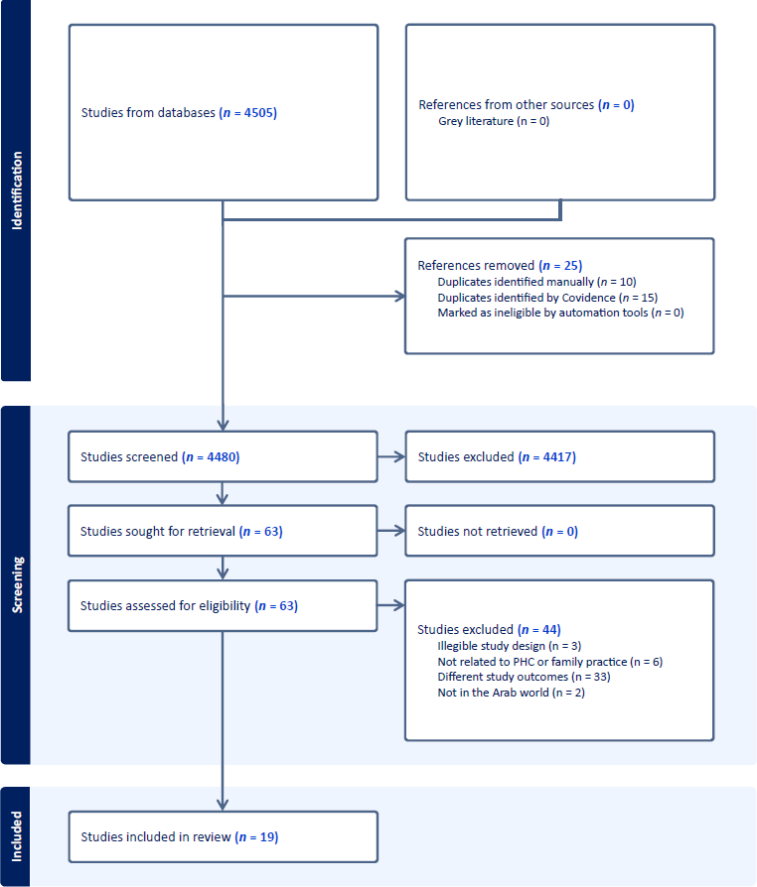
Preferred Reporting Items for Systematic Reviews and Meta-Analyses (PRISMA) flow diagram of study selection. PHC = primary health care

As shown in the data presented in Figure 1, the searched databases yielded 4505 studies and 4480 studies were screened following the removal of 25 duplicate entries. The dataset was subsequently filtered based on the criteria of title and abstract, removing 4417 studies. Out of the total of 63 studies, the researchers retrieved and assessed their eligibility. Among these, 44 studies were deemed ineligible and subsequently rejected. Expressly, 33 reports were excluded based on their out-of-scope measurements. Six studies were excluded from the analysis because they failed to evaluate FM or primary care settings. Additionally, three studies were excluded because their study designs did not allow for concluding perceptions of FM or PHC, and two studies were excluded as their population did not originate from the Arab world. The total number of articles incorporated in the study was 19.

The majority of the studies had a moderate risk of bias, five studies had a low risk of bias, and only one study had a high risk of bias; this latter study was included in the analysis to retrieve potential outcomes (Supplementary Box I).

Most studies employed a cross-sectional design, while two utilised a qualitative approach and one used a mixed-methods design. Some cross-sectional studies utilised an online form in addition to self-reported questionnaires. Ten studies were conducted in Saudi Arabia, two in Egypt, three in Iraq, two in Jordan, one in Oman, and one in Morocco. Numerous studies failed to disclose their funding sources and potential conflicts of interest, as seen in Supplementary Table 1.

The 19 studies targeted different types of populations. Seven studies targeted university students and house officers, two studies targeted physicians, five studies targeted the general population, and six studies targeted PHC clients.

Table 1 summarises the results of studies on students and house officers. Medical students had better knowledge and perception than non-medical. Medical students were aware of the role of family doctors as gatekeepers, delivering holistic and continuity of care. FM was not a preferred career path among medical students owing to concerns regarding its prestige and salary; reasons given by those who were positive about FM included the opportunity for work–life balance in family practice and being an exciting specialty from a research perspective.

**Table 1.** The studies on medical students and the main results

Study ID	Title	Inclusion	Studies' findings
Desouky *et al* 2018^29^	Saudi university students’ awareness and attitude towards family medicine specialty	All students who agreed to participate in the study from the two colleges at Taif University	Medical students in Saudi Arabia had better knowledge and perceptions of FM than non-medical students but were less interested (6%) in choosing it as a future career. They were concerned about its low status within the medical profession, limited scientific prestige, and lower salary.
Sebbani *et al* 2020^30^	Medical students' career choice and attitudes towards family medicine in Morocco	Students in the first cycle (first and second year of medicine) or second cycle (from the third year until obtaining the medical degree)	Medical students in Morocco had a positive perception of general practice but had a low interest in becoming a GP as a future career (6.4%) owing to less prestige and low compensation. It was found 27.5% would choose to become a GP if it were an actual specialty (FM, as part of the new medical reform). The study recommended early exposure to FM concepts and specialists and increasing the frequency of contact with competent GPs in the context of strategies for upgrading general medicine among undergraduate students.
Alshammari *et al* 2019^31^	Attitude of medical students at King Saud Bin Abdulaziz University for Health Sciences toward family medicine as a future specialty	Medical students in the third through to sixth years in King Saud bin Abdulaziz University for Health Sciences (KSAU-HS), College of Medicine, Riyadh, Saudi Arabia	Most students (74.3%) were aware of the importance of FM, including aspects such as being well respected (45.2%), more opportunity for work–life balance (69.8%), focusing on prevention (77.6%), building long-term relationships (85%), whole-person focus (84.8%), and gatekeeper function (68.9%). There was no significant difference between the clinical and pre-clinical students or difference related to sex. However, only a few of them (2.6%) planned to choose the FM specialty. The study did not examine attitudes toward FM as a future career. The study found participants agreed strongly that FM was diagnostically challenging (31.5%), as exciting as other specialties (19.5%), and received the same amount of training as other specialists (29.5%).
Elkhawaga *et al* 2015^32^	House officers' attitude towards family medicine and its choice as a career in Egypt	All HO (437) registered to attend this voluntary training course in FM	The study found 51.5% considered FM to have an essential social function, was an exciting specialty from a research perspective (43.8%), and provided a high salary compared with other specialties (20.9%). The most influential factor in HOs’ opinions regarding FM was their own experiences during the study (52.6%). More than two-fifths of HOs reported that training sessions in health centres would be helpful, and 29.5% stated that at least 25%–50% of the total practical training in medical school should be dedicated to FM. The most typical reasons for choosing FM as a career were benefit to patients (72.5%), working hours (53.9%), working lifestyle (51.8%), and financial reasons (49.9%).
AlKot *et al* 2015^33^	Family medicine in Egypt from medical students' perspective: a nationwide survey	Undergraduate medical students in Egypt in the academic year 2012–2013 (approximately 50 000 students)	Medical students in Egypt positively perceived FM as an essential specialty but had little interest in its choice as a future career. Only 4.7% chose FM as a career. Factors associated with FM choice included females, good knowledge of FM, and the belief that FM benefits Egypt.
Alyousefi 2017^34^	Knowledge and attitude of Saudi medical students towards the family medicine specialty during their family medicine course and its effect on their career plans: a comparative study	Fourth-year medical students who were in the family medicine course	The top reasons why students included FM in their career plans were observations of the physician–patient interaction in this specialty (66.7%), the faculty staff’s attitudes, interests, and compassion (61.5%), and the enjoyment of the FM rotation (51.3%). Based on the study findings that the FM clinical rotation (taught since 1980), which includes 4 weeks didactics and clinical attachments in PHC, was highly beneficial for medical students in terms of improving their knowledge about and attitude toward the field, the choice of FM as a career was not affected by the course. Instead, the student’s perception and passion to pursue FM as a practice influenced the student’s career choice.

FM = family medicine. HO = house officer

Table 2 provides a summary of findings from two research studies conducted on physicians. One of the studies focused on physicians from various specialties, including emergency medicine, obstetrics and gynaecology, paediatrics, internal medicine, surgery, psychiatry, ophthalmology and orthopedics; the other study targeted primary care physicians. Surgeons in obstetrics and gynaecology and medicine were least satisfied with the role of family doctors in the community in Saudi Arabia. Meanwhile, GPs in Iraq agreed on the role of family doctor as the gatekeeper.

**Table 2.** Studies on physicians and the main results

Study ID	Title	Inclusion	Studies' findings
Alzahrani *et al* 2020^19^	Perception toward the family medicine services among the physicians of Prince Sultan Military Medical City, Riyadh City, 2018: cross-sectional study	Physicians who were actively practising and working at Prince Sultan Military Medical City under the departments of internal medicine, surgery, psychiatry, emergency medicine, paediatrics, obstetrics and gynaecology, ophthalmology and orthopedics. Physicians who had job titles of registrars, senior registrars, and consultants. Both sexes, no age, or nationality restrictions	Physicians expressed good perceptions of FM practice; psychiatrists, ophthalmologists, and paediatricians were most satisfied. Surgeons in obstetrics and gynaecology and medicine were least satisfied. Most departments saw the need for FM and its importance for improved communication, such as better referral letters between departments.
Salman *et al* 2017^18^	The attitude of a sample of general physicians working in some primary health care centers in Baghdad, Al-Karkh, towards family medicine	General physicians in 20 primary health care centres in Baghdad, Al-Karkh directorate	Most GPs in PHC (90%) scored high, responded positively, and agreed overall about the role of family medicine in Iraq. The study found 97% agreed that FM is the point of first medical contact within the healthcare system.

FM = family medicine. PHC = primary health care

As Table 3 shows, Saudi Arabia studies were favourable in assessing the community’s perception of the role of family doctors. Most had heard about FM. However, most did not have regular family physicians, nor did they know where FM practices were located or even understand the difference between a family doctor and a GP.

**Table 3.** Studies on the general population and the main results

Study ID	Title	Inclusion	Studies' findings
AlBarrak *et al* 2019^35^	Public awareness and perception about the role of family physicians in Saudi Arabia: a cross-sectional study	Able to understand and write in Arabic; aged ≥18 years; resident in Jeddah, Saudi Arabia (JSA), and giving written consent for completing an online survey	The general population of JSA had moderate positive responses towards the family physician’s role as a vital element of the healthcare system. Still, most (62%) had never visited a family physician, and more than half (57%) didn’t know where clinics were located.
Alshammrani *et al* 2022^36^	Public awareness and perception of family medicine in Jeddah, Saudi Arabia	Adults aged ≥18 years living in Jeddah, Saudi Arabia	The general population of Jeddah had heard of FM (86.7%) and had moderate positive responses towards the role of a family physician (55.1%) as a vital element in the healthcare system. However, most participants (61.7%) had never previously visited a family physician and were unfamiliar with the locations of FM clinics (57.2%). Therefore, the participants called for public education on FM’s role in therapeutics and preventive services.
Aldhamadi and Alzahrani 2019^25^	The public perception of and attitude toward primary healthcare centers in comparison to other specialties among the Saudi community	Saudis aged ≥18 years who attended health campaigns in two different shopping malls in the same region in Saudi Arabia	The study found 66.8% of the responders preferred PHC as their first choice to address a medical issue. Approximately 60% of the responders agreed that PHC generally provided quality care. While 54.5% of the responders confirmed that PHC provided them with easy access to the healthcare system through first-contact services. A higher percentage of the study population believed the ED had a more critical role in the healthcare system than the FM department. Participants would seek care in the ED first for concerns such as a dog bite, smoke inhalation after a house fire, and a complete hand burn. The ED choice was owing to long waiting times in PHC and the convenient locations of the ED.
Elagi *et al* 2019^13^	Public’s perception and satisfaction on the role and services provided by family physicians in Saudi Arabia: A cross-sectional study	Aged ≥18 years, and residing in the Jazan region, KSA, for at least 6 months	Most (90%) did not have a regular family physician. While 81.2% were aware of the family physician principles, 73.3% of the essential family physician role, 59.9% were aware of the conditions family physicians treat, and 76.7% agreed on the value of family physicians involved in their care. The study found 59.8% preferred to see a specialist other than FM first; reasons were not identified.
Murad *et al* 2022^37^	Community perspective on family medicine and family physician in Saudi Arabia 2020	All Saudi Arabia residents who agreed to participate in the study were aged ≥15 years. There were no restrictions on sex, nationality, occupation, residence, or the socioeconomic level of the participants	The general population in Saudi Arabia was aware of the importance of family physicians, and they trusted them. The study found 56.6% did not understand the difference between FM and GP. Patients’ perceptions, whether awareness and knowledge or experience and attitude scores, were significantly correlated (*P*<0.001) to chronic illness status, being a healthcare worker, job, marital status, and sex factors. Moreover, the experience and attitude score was additionally correlated to residence region, central (Spearman’s rho = 0.03; *P* = 0.034) and participants’ nationality, Saudi (Spearman’s rho = 0.07; *P*<0.001).

ED = emergency department. FM = family medicine. PHC = primary health care

Primary care clients showed more satisfaction with family doctors than GPs because of their better communication and clinical skills. Having a committed doctor who clarifies issues and informs patients about management plans is important, whether the doctor is an internist or a family doctor (Table 4).

**Table 4.** Studies on primary care clients and the main results

Study ID	Title	Inclusion	Studies' findings
Barghouti *et al* 2019^38^	Exploring agendas of patients attending family medicine clinics in Jordan. A qualitative content analysis study	Patients aged >18 years attended the FMC at the University of Jordan, Amman, Jordan	FM patients' main concerns were receiving treatment for an acute illness (33.8%), followed by the desire to clarify health conditions (25%). The study found 40% believed that a health condition rather than lifestyle caused their symptoms, while 32.5% had no speculations about their causes. In addition, 36.3% expected doctors to provide information related to their health condition. While 29% had concerns about administrative and structural issues.
Boubshait *et al* 2022^39^	Patient trust in primary care physicians: a mixed methods study of persons with diabetes at university-based clinics in the Eastern Province of Saudi Arabia.	Adult patients with diabetes, both sexes between the ages of 18 years and 65 years, who were attending the internal medicine (IM) or family medicine (FM) clinics at King Fahad Hospital of the University (KFHU) or its Family and Community Medicine Center clinics in Eastern province of Saudi Arabia	Patients' trust in primary care IM and FM physicians was similar. The most important factor was having a respectful, committed doctor who clarifies, engages, and informs the patient of the management plan. Competence, comprehensiveness of care, and the physician’s ethical conduct and personal traits were also important.
Al-Dabbagh and Hassoon 2020^40^	Comparison of clients’ satisfaction in family health adapted centers and primary health care centers in Baghdad 2018	Community participants aged ≥18 years who had at least two visits to the included centres	Overall, patients’ satisfaction in family health adapted centres was higher than in traditional primary health care centres in all the studied dimensions, reflecting the better quality of care in family health adapted centres.
Ismael *et al* 2022^41^	Inhabitance comprehension about family physicians who are working in a family medicine Tariq Baghdad center at Kirkuk city	Aged ≥18 years; clients of the Tariq Baghdad family medical center in Kirkuk city	Patients in the FM clinic, both male and female, were delighted with the care. Participants who were statistically more satisfied were older, less educated women. Preference for seeing the same physician was 66.67%, and FM was preferred over GP, 84.3% versus 7.14%.
Al-Kindi *et al* 2019^42^	Patients’ perceptions of communication and clinical skills of primary healthcare physicians in Oman	Omani patients who were aged ≥18 years in 12 PHC centres	Patients favourably viewed specific skills in communication and clinical aspects of family physicians compared with GPs. Family physicians received better responses for almost all items but without reaching statistical significance. Family physicians received 4 years of residency training beyond GPs.
Alnawafleh *et al* 2022^43^	Experiences of primary health care clients in Jordan: qualitative study	Recruitment was a purposeful selection from primary care attendants or clients at PHC service providers from the Ministry of Health (MoH) clinics and the private sector into focus groups at three different geographic locations.	There was comfort and full access to PHC service. Clients were sometimes unsatisfied owing to the absence, inadequacy, and poor quality of the service. This may lead to several visits without getting the service required. Authors called for training beyond GPs for PHC.

FM = family medicine. FMC = family medicine clinic. IM = internal medicine. PHC = primary health care

## Discussion

### Summary

Nineteen studies met the criteria to be included in this review about the perceptions of the specialty of FM in Arab countries. Participants in these studies included medical students (five) and house officers (one), physicians (two), patients (PHC clients; six), and the general public (five). Overall, there was a positive perception of FM and the specialty’s role as a first point of contact or gatekeeper, managing chronic health issues and promoting preventive care. Despite the positive opinions, most responders in the general population studies had not encountered a FM physician or were confused about the difference between a GP and an FM physician.

The two studies that surveyed physician colleagues showed positive regard from GPs and colleagues in other specialties (Salman *et al*, 2017 and Alzahrani *et al*, 2020).^18,19^ While medical students were aware of FM and its role in healthcare delivery and reported finding FM rotations valuable, less than 5% of the students in any of the studies considered it a career choice. Reasons included its low status within the medical profession, limited scientific prestige, and lower salaries. However, house officers and students wanted more exposure to FM early on in their training.

### Strengths and limitations

The majority of the studies reviewed exhibited low quality. Only one study employed an instrument characterised by a low risk of bias. This was achieved using a representative sample and a random sampling strategy while minimising non-response. Furthermore, it is worth noting that certain studies have solely focused on a single geographic location, recruited participants exclusively through health centres, or targeted specific medical schools. This approach may have inadvertently added selection bias to the research findings. In addition, social desirability bias may have influenced the optimistic view of FM as questionnaires or interviews were implicitly or explicitly based on a positive view of FM. Hence, there is a lack of clarity on the correct measurement of constructs and the generalisability of findings to the broader population in the analysed countries. Therefore, it is imperative to do rigorous research to understand the variations in how Arab communities perceive the field of FM and its practice on an international scale. Specifically, there is a requirement for qualitative studies that explore various constructs related to this topic. Given that only two qualitative studies and one mixed-method study fulfilled the inclusion criteria, it is imperative to conduct additional qualitative research to obtain a more comprehensive understanding of Arab communities' perspectives on family medicine specialisation and practice. Such research has the potential to illuminate health systems and medical and educational factors that are modifiable and thus may necessitate consideration to improve the practice in this particular medical discipline.

### Comparison with existing literature

FM programmes in the Middle East and North Africa were established after adopting the Declaration of Alma-Ata in 1978. Lebanon, Bahrain, and Israel were the first countries to develop FM residency programmes. Other postgraduate programmes were established in the 1980s (Kuwait, Jordan, Turkey), in the 1990s (Qatar, United Arab Emirates, Oman, Saudi Arabia), in the 2000s (Iran), and more recently in 2010 (Palestine) and 2011 (Tunisia). A 2011 survey of FM residency programmes in Arab countries found 31 programmes graduating only 182 residents per year, a low ratio of FM to the population.^5^ The WHO astern Mediterranean Region and World Organization of Family Doctors (WONCA) publication shows some progress but woefully inadequate numbers to meet the need for adequate primary care access.^6^

The limited interactions with FM physicians and the limited experience and interest in the medical school reported in our review are most likely a result of the scarcity of family physicians and the absence of FM in medical school curricula. Oman, which has one of the most established FM-based health systems, acknowledged the importance of FM in the healthcare system,^20^ and stressed the need to retain and attract more FM physicians. This can be done by ensuring that primary healthcare centres have adequate laboratory capabilities and medications and that they use team-based care approaches so that patients with non-communicable diseases can be managed in primary healthcare centres. Legislation should link populations to a primary healthcare catchment area. Robust primary health care ensures that patients’ needs are met and avoids seeking secondary and tertiary services without first accessing primary care.^21^ It also provides adequate support so that family physicians can make use of the full range of their skills.

Perceptions and attitudes towards FM vary widely among medical students, practising physicians, patients, and the general public. Understanding these perceptions is crucial for addressing challenges in recruiting and retaining FM practitioners and improving the healthcare delivery system. Medical students' attitudes towards FM are influenced by various factors, including curriculum exposure, mentorship, and personal experiences.^22,23^ Studies indicate that early and positive exposure to FM can significantly enhance students' interest in this field. Studies from Saudi Arabia have shown a positive perception of FM among medical students as there is good curriculum exposure in medical education there. Medical students with substantial primary care clerkship experiences were more likely to pursue careers in FM.^22^ However, persistent stereotypes and misconceptions about FM negatively impact student interest. Many students perceive FM as less prestigious than surgery or cardiology. Concerns about lower income and perceived lack of specialisation also deter students from choosing FM as a career path.^23^

Practising family physicians often report high job satisfaction owing to the variety and continuity of care they provide. They appreciate the holistic approach to patient care and the opportunity to build long-term relationships with patients. However, challenges such as administrative burdens, lower reimbursement rates compared with specialists, and high patient loads can lead to burnout and job dissatisfaction.^24^

Patients generally hold family physicians in high regard owing to their accessibility, comprehensive care, and personal touch.^25^ Studies have shown that patients value the trust and continuity of care provided by family physicians, contributing to better health outcomes and patient satisfaction.^26^ However, our results show that some patients may perceive family physicians as less specialised and prefer consulting specialists for specific health issues. This perception can be influenced by the growing emphasis on specialisation within the healthcare system and the belief that specialists offer more advanced care for complex conditions.^27^

The general public’s perception of FM is shaped by media representation, personal experiences, and broader societal attitudes towards primary care. FM is often viewed positively for providing accessible and patient-centred care. Nevertheless, there is a lack of understanding of the full scope of FM, which can lead to an underappreciation of the specialty’s complexity and the comprehensive training required for family physicians.^28^

### Implications for research and practice

Our review findings suggest the following recommendations: (1) an education campaign for the general public about the role of FM; (2) increasing training capacity for family physicians by increasing residency slots; (3) early exposure to family physicians during medical school and including FM in the curricula; (4) developing a process for continually improving the education and quality of family physician practice so that they can incorporate the rapid changes in science into the care they provide; and (5) further research on the challenges to FM practice in Arab countries to understand the situation better and work towards its improvement.

Other countries with longstanding training in the specialty of FM face similar challenges with engaging medical students.^23,24,26^ Research in these settings underlines the importance of early exposure of medical students to practising family physicians, including FM in medical school curricula, and family physicians engaged in research so that students understand the full range of family physicians' skills.

## Trial registration number

The research was registered in PROSPERO (Reg ID CRD42023387889).

## Data

Data can be obtained from the corresponding author upon request.
